# Editorial: Recent progress in drug delivery technologies for advanced pharmaceutics

**DOI:** 10.3389/fchem.2024.1413056

**Published:** 2024-04-24

**Authors:** Bing Guo, Tingting Peng, Qinglian Hu, Ruirui Qiao

**Affiliations:** ^1^ Shenzhen Key Laboratory of Flexible Printed Electronics Technology, Shenzhen Key Laboratory of Advanced Functional Carbon Materials Research and Comprehensive Application, School of Science, Harbin Institute of Technology, Shenzhen, China; ^2^ State Key Laboratory of Bioactive Molecules and Druggability Assessment, Jinan University, Guangzhou, China; ^3^ College of Biotechnology and Bioengineering, Zhejiang University of Technology, Hangzhou, China; ^4^ Australian Institute of Bioengineering and Nanotechnology, The University of Queensland, Brisbane, QLD, Australia

**Keywords:** nanomecidines, biomaging, theranostics, diseases, therapy

The incidence of many diseases like tumors, neurodegenerative diseases, bacterial infections, inflammation, and cardiovascular diseases have increased over the last decade. Despite the progress in invasive treatment paradigms including surgery and radiotherapy, drug mediated minimal/non-invasive therapies are still in the main stage. However, owing to the physiological and pathological barriers, medicines always show limited accumulation and retention in disease tissue, nonnegligible side effects, and even multiple drug resistance, leading to minimized therapeutic outcomes. Increasing attention has been devoted to investigating advanced drug delivery systems and technologies to across the barriers, increase targeting capability, and improve pharmacokinetics (PK) and pharmacodynamics (PD). To achieve efficient drug delivery, the key is to customize drug delivery technology to specific diseases and even individual patients for precision treatment. It is a great honor that in Frontier in Chemistry we launch this Research Topic, “Recent Progress in Drug Delivery Technologies for Advanced Pharmaceutics,” which would include the state-of-the-art drug delivery technologies, which hold promise to overcome the physiological and pathological barriers, mitigate drug accumulation and retention in disease tissue, and optimize the treatment outcomes. Importantly, both reviews and original research articles are included in this Research Topic.

Since smart drug delivery systems could alleviate the un-wanted drug release during circulation, it would lower-down the therapeutic threshold dosage, and improve prognosis, with potential to be next-generation of clinic drug delivery systems. Farjadian et al. reviewed the recent development of physically triggered nano-systems (PTNSs) based on polymeric micelles and hydrogels, mesoporous silica, and magnets, which are mainly used for diagnostic or therapeutic and theranostic applications. The authors put special focus on PTNSs which can respond to internal or external stimuli, and illustrate how to develop and optimiz stable, precise, and selective therapeutic or diagnostic agents, for meeting the requirements of efficacy, toxicity, pharmacokinetics, and safety before clinical investigation (Farjadian et al.).

The efficiency for delivery of clustered regularly interspaced short palindromic repeat/CRISPR-associated protein (CRISPR/Cas) systems still suffers from limited carriers which assist the drug to execute genome-editing functions. So far, researchers have devoted tremendous efforts in this area, and the timely summary would expedite further development and trigger interest among wide audience. Thus, Hejabi et al. reviewed the state-of-the-art drug delivery systems for clustered regularly interspaced short palindromic repeat/CRISPR-associated protein (CRISPR/Cas), including both viral nanocarriers and non-viral carriers like liposomes, polymeric, and gold particles.

As osteosarcoma is one of the most lethal cancer and there is a lack of efficient drug carriers customized for treating osteosarcoma, Deng et al. developed a pH sensitive charge-conversion polymeric micelle [mPEG-b-P(C7-co-CA) micelles] for osteosarcoma-targeted delivery of cinnamaldehyde (CA). Their results showed that the nanosystem holds great promise to reduce the risk to patients and improve survival rates (Deng et al.).

Nowadays, colitis inflammation has attracted increasing attention and is known as trigger for many diseases, while the key to treat this colitis inflammation is usage of efficient drug carriers. Recently, Chang et al. designed an antioxidant coordination polymer nanoparticle using the engineering of procyanidin (Pc) and free iron (Fe), named Pc-Fe nanozyme, for effectively scavenging ROS and further inhibiting inflammation while altering the gut microbiome for the treatment of colitis. They showed their nanoparticles are promising candidates for clinical translation on IBD treatment and other ROS induced intestinal diseases (Chang et al.).

Systemic lupus erythematosus (SLE) is a well-known autoimmune disease, which is difficult to treat so far. To meet up this challenge, Zhang et al. developed a hollow polydopamine (HPDA) nanocarrier loaded with CTX by ionization to prepare the novel nanoplatform, CTX@HPDA, for dual photothermal and chemotherapeutic treatment of diffuse alveolar hemorrhage (DAH) which is a serious complication caused by. Their results demonstrated that the dual therapeutic nanoplatform is a promising candidate for disease treatment and offered insights for scientists to further develop functional therapeutic platforms (Zhang et al.).

As editors, we hope this Research Topic would embark interest among wide audience and serve as spark to novel ideas and efforts for researchers with different backgrounds.

